# Biomarkers for Zoonotic Visceral Leishmaniasis in Latin America

**DOI:** 10.3389/fcimb.2018.00245

**Published:** 2018-07-26

**Authors:** Claudia I. Brodskyn, Shaden Kamhawi

**Affiliations:** ^1^Instituto Gonçalo Moniz, Fiocruz Bahia, Salvador, Brazil; ^2^National Institute of Allergy and Infectious Diseases (NIAID), National Institutes of Health, Bethesda, MD, United States

**Keywords:** zoonotic visceral leishmaniasis, *Leishmania* infantum, biomarkers, cytokines/chemokines, canine visceral leishmaniasis, human visceral leishmaniasis

## Abstract

In Latin America, zoonotic visceral leishmaniasis (ZVL) arising from infection by *L. infantum* is primarily transmitted by *Lutzomyia longipalpis* sand flies. Dogs, which are chronic reservoirs of *L. infantum*, are considered a significant risk factor for acquisition of ZVL due to their close proximity to humans. In addition, as a vector-borne disease the intensity of exposure to vector sand flies can also enhance the risk of developing ZVL. Traditionally, IFN-γ and IL-10 are considered as the two main cytokines which determine the outcome of visceral leishmaniasis. However, more recently, the literature has demonstrated that different mediators, such as lipid mediators (PGE-2, PGF-2 alfa, LTB-4, resolvins) and other important inflammatory and anti-inflammatory cytokines are also involved in the pathogenicity of ZVL. Analysis of a greater number of mediators allows for a more complete view of disease immunopathogenesis. Additionally, our knowledge has expanded to encompass different biomarkers associated to disease severity and healing after specific treatments. These parameters can also be used to better define new potential targets for vaccines and chemotherapy for ZVL. Here, we will provide an overview of ZVL biomarkers identified for both humans and dogs and discuss their merits and shortcomings. We will also discuss biomarkers of vector exposure as an additional tool in our arsenal to combat ZVL.

## Introduction

Leishmaniasis is considered a neglected tropical disease with approximately 350 million people at risk of infection, and with 2 million new cases reported annually, mainly in extremely impoverished communities (WHO/Leishmaniasis, 2014). The clinical manifestations of leishmaniasis range from cutaneous ulcers to the visceral form, one of the most severe which can be fatal if left untreated (Desjeux, 2004). Over 90% of visceral leishmaniasis (VL) cases worldwide are concentrated in six countries: India, Bangladesh, Sudan, South Sudan, Ethiopia, and Brazil. VL arises from either *L. donovani* in the Indian subcontinent and East Africa, or *L. infantum* in Europe and Latin America, and kills 20,000–40,000 people annually throughout the world (Alvar et al., 2012).

Dogs are considered the main reservoirs of *L. infantum* parasites and the presence of these animals in endemic areas represents a risk factor for human disease. The disease in dogs shares characterisitcs with human VL, providing a good model to study the immunopathogenesis of *L. infantum* infections. Canine visceral leishmaniasis (CVL) is also of veterinary interest since the disease is spreading to big cities in Latin America including Belo Horizonte, São Paulo, Natal, and Camaçari (Alves and Bevilacqua, 2004), threatening public health.

*L. infantum* multiplies inside macrophages in the liver, spleen and bone marrow. In human VL, 90% of infected individuals remain asymptomatic or subclinical, showing an intense cellular immune response characterized by a positive delayed-type hypersensitivity reaction to *Leishmania* antigens. However, in patients who progress to clinical disease, an enlargement of the spleen and liver may be observed, accompanied by hematological disorders, notably anemia, thrombocytopenia, which may result in hemorrhaging, and neutropenia. These disorders can increase host susceptibility to bacterial infection and patients with VL often suffer weight loss or fever (Werneck et al., 2003).

The search for biomarkers for prognosis of human and canine VL and measurement of the success of treatment has intensified in the last few years. This led to a better knowledge of disease immunopathogenesis and favored therapeutic and prophylactic strategies for ZVL. As *L. infantum* is transmitted by the bite of phlebotomine sand flies, several markers of vector exposure have also been identified and used as tools to assess success of interventions. Used in combination, biomarkers of disease and vector exposure can provide powerful tools to support efforts to control ZVL.

## Human ZVL

The progression of ZVL is associated with immunosuppression, characterized by lack of a cell-mediated immune response to *Leishmania* antigens. Accordingly, the absence of lymphocyte blastogenesis and IFN-γ production have been associated with progression to VL (Carvalho et al., 1992). Interestingly, patients become responsive to *Leishmania* antigens after successful therapy (Carvalho et al., 1989). Biomarkers were evaluated in a group of ZVL patients given standard antimonial treatment (Schriefer et al., 1995). Soluble CD4 (sCD4), sCD8, and sIL-2R levels were higher in sera of patients compared to healthy controls. After treatment, levels of the above-mentioned biomarkers exhibited a significant decrease in patients who responded to therapy. Importantly, when comparing pretreatment levels of these markers among those who responded to antimonial therapy and refractory patients, the serum concentrations of sCD8, sIL-2R as well as neopterin were significantly elevated in refractory patients. Therefore, these markers represent promising indicators of a patient′s response to antimonial therapy (Schriefer et al., 1995).

The cure for human ZVL is associated to induction of a Th1 immune response characterized by IFN–γ production. IFN–γ has an essential role in controlling the parasite load and in the development of a long-lasting immunity. Asymptomatic patients also exhibit a Th1 response, suggesting that IFN-γ activates macrophages, increasing their leishmanicidal ability and maintaining the infection under control (Kaye and Scott, 2011). In contrast, anti-inflammatory cytokines, mainly IL-10, lead to proliferation of parasites and interfere with infection control (Nylen and Sacks, 2007; Gautam et al., 2011). In a study of patients with active ZVL, *in vitro* stimulation of PBMC with *Leishmania* antigens showed an inverse pattern with low levels of IFN-γ during active disease that augmented steadily after treatment (Caldas et al., 2005). Interestingly, these patients showed elevated plasma levels of IFN-γ, IL-12p40, and IL-10 during active disease that sharply decreased after treatment (Caldas et al., 2005). Therefore, IFN-γ and IL-10 are the main hallmarks of infection by *L. infantum*, and the balance between these cytokines seems to be essential for control of the infection.

IL-17, a cytokine produced mainly by Th17 cells, is known for inducing the production of chemokines that recruit neutrophils to inflammatory sites. A cohort of individuals with VL caused by *L. donovani* showed that IL-17 seems to be protective (Pitta et al., 2009). However, in ZVL patients caused by *L. infantum*, high levels of this cytokine did not induce IFNγ/NO in enough concentrations to lead to a recovery from disease (Nascimento et al., 2015).

In severe forms of ZVL, there is an exaggerated inflammatory response that leads to disseminated intravascular coagulation and other manifestations such as hemorrhage (Costa et al., 2013). Children displaying an intense production of cytokines have a higher risk of death. IL-6 seems to be one of the main cytokines associated with fatal disease, but IFN-γ, IL-1β, IL-8, and TNF-α have also been associated to ZVL severity (Costa et al., 2013). In fact, *L. infantum* may activate inflammatory reactions via CD14, leading to a production of several cytokines such as IFN-γ, IL-27, IL-10, IL-6 as well as sCD14 (Dos Santos et al., 2016). These data reinforce previous results and denote the interdependent relationship between pro-inflammatory (IFN-γ, IL-6 and TNF-α) and anti-inflammatory (IL-10 and IL27) cytokines. The authors also showed that higher levels of IL-6 (>200 pg/ml) are associated to death (Dos Santos et al., 2016). Collectively, this highlights the complexity of finding a good biomarker for ZVL since induction of a cytokine such as IFN-γ may be associated to protection or severe disease, depending on its levels and the overall inflammatory environment.

Other biomarkers, including lipid mediators, have been associated with ZVL and could also be used to monitor the efficacy of specific therapies. Araujo-Santos et al. (2017) reported a distinct biosignature of active ZVL through increased serum levels of Prostaglandin F 2 alfa (PGF_2α_), Leukotrine B4 (LTB_4_), Resolvin D1 (RvD1), TNF-α, IL-1β, IL-6 and IL-8, IL-10, and IL-12p70, as well as decreased concentrations of TGF-β1 in comparison to healthy endemic controls, regardless of patient age or gender. Following the onset of leishmanicidal treatment, the inflammatory cytokine profile, as well as the relationships between these markers and several hematological and biochemical parameters, gradually reverted, which suggested that the observed cytokine expression profile was induced by active disease or infection (Araujo-Santos et al., 2017). Of the quantified markers, TGF-β1 concentrations were significantly elevated, while IL-6, IL-8, IL-10, and RvD1 levels substantially decreased, after 30 days of therapy in comparison to their levels during active ZVL infection.

It is worthwhile noting that common immunological signatures were observed in sera of VL patients from Brazil and Bangladesh infected with *L. infantum* and *L. donovani*, respectively. Inflammatory and regulatory cytokines (IFNγ, TNFα, IL-10, IL-17), as well as levels of growth factors (FGF-fibroblast growth factor; VEGF, vascular endothelial growth factor), were elevated in the serum of VL patients from both regions (Duthie et al., 2014). Serological assays from Brazilian patients obtained during and after meglumine antimoniate treatment demonstrated that multiple parameters reverted to concentrations similar to healthy endemic controls. The authors suggested that a multi-parameter signature of the response to treatment could be useful in clinical trials to evaluate the success of therapeutic interventions (Duthie et al., 2014).

Macrophages infected by *L. infantum* present an M2b-like phenotype as well as a C-type lectin receptor (CLR) signature, characterized by Dectin-1, mannose receptor and DC-SIGN homolog SIGNR3 expression (Lefevre et al., 2013). Expression of Dectin-1 and the mannose receptor are essential to the leishmanicidal effect of macrophages, leading to the production of ROS and also the induction of IL-1β secretion. On the other hand, SIGNR3 was shown to favor parasite survival via the inhibition of the LTB-4-IL1β axis (Lefevre et al., 2013).

More recently, MCP-1 was shown to be a good biomarker to identify asymptomatic individuals infected by *L. infantum* (Ibarra-Meneses et al., 2017). This chemokine is expressed 110 times more strongly than IL-2 in cultures of whole blood stimulated with *Leishmania* antigens, identifying 87.5% of asymptomatic subjects; it is also significantly increased in all patients cured of ZVL (Ibarra-Meneses et al., 2017). Table 1 summarizes the main findings about biomarkers in human ZVL.

**Table 1:** Biomarkers for human visceral leishmaniasis.

**Cytokines**	**Chemokine**	**Lipid mediators**	**Mixed molecules**	**Signature**	**References**
IFN-γ, IL-12, and IL- 10 TNFα, IL-17			sCD4, sCD8, and sIL2R	↑ Active disease	Schriefer et al., 1995; Gautam et al., 2011
				↓ Post-treatment	Nylen and Sacks, 2007; Duthie et al., 2014; Araujo-Santos et al., 2017
			sCD8, sIL-2R, neopterin	↑ Patients refractory to treatment	Caldas et al., 2005
	MCP-1			↑ Asymptomatic VL ↑ Cured patients	Kaye and Scott, 2011; Ibarra-Meneses et al., 2017
IL-6				↑ Fatal Disease (higher risk of death)	Costa et al., 2013; Dos Santos et al., 2016
IL-1β, IL-8, and TNF-α, IFN-γ, IL-27, IL-10				Severity of disease	Dos Santos et al., 2016
			sCD14	↑ Severe disease	Dos Santos et al., 2016
IL-1β, IL-6 IL-8 TGF-β1		PGF2α, LTB4, RvD1		↓ Active ZVL Post-treatment ↓ Active ZVL ↓ Post-treatment	Araujo-Santos et al., 2017
–			FGF, VEGF	↑ Active VL ↓ Post-treatment	Duthie et al., 2014

In summary, biomarkers other than IFN-γ and IL-10 have been described more recently that reveal the complexity of ZVL. These molecules have demonstrated both their value as biomarkers of disease progression and their usefulness in monitoring the efficacy of treatment. In the future, such biomarkers may also be of value in assessing the level of protection induced by prophylactic strategies.

## Canine VL

Dogs are one of the main urban reservoirs of *L. infantum* parasites and their presence in endemic areas is a risk factor for the development of human disease, due to their role in propagating infection in phlebotomine sand flies. Clinical manifestations of CVL present a wide spectrum of clinical signs that are non-specific. However, a high proportion of animals do not progress to disease, control the parasites and live for years or their entire life without any clinical signs (Foglia Manzillo et al., 2013). The presence of these dogs in the endemic area contributes to maintenance of the parasites, since they can transmit *L. infantum* to the sand flies. The resistance or susceptibility to CVL is directly correlated with the induction of either a Th1 response characterized by IFN- γ, IL-2, and TNF-α production, or a Th2 response with the production of IL-4, IL-5, IL-10, IL-13, and TGF-β, respectively, and the level of immune activation is considered to directly influence disease severity (Reis et al., 2010; Barbosa et al., 2011). In fact, a reduction in the burden of *L. infantum* was related to elevated expression of IFN-γ and TNF-α, whereas increased IL-10 and iron regulatory protein 2 (IRP2) expression and an increase in plasma albumin levels were associated with a higher parasite burden (do Nascimento et al., 2013). Accordingly, the dynamics between different aspects of the immune response and intracellular iron availability could play some role in the evolution of *Leishmania* infection (do Nascimento et al., 2013).

The predominance of a Th2 response in infected dogs leads to the appearance of M2 macrophages, identified by CD163 immunostaining, mainly in the spleen, muzzle, ear and popliteal and pre-capsular lymph nodes (Moreira et al., 2016). The highest proportion of M2 macrophages coincided with the highest parasite loads and were found in more susceptible organs of infected dogs such as the spleen and lymph nodes, as well as skin, considered a more resistant organ. In contrast, the liver showed low parasitism and weak immunostaining for M2 macrophages that was not significantly different between infected and negative groups of dogs. Therefore, M2 macrophages may contribute to parasite proliferation in organs (Moreira et al., 2016). Another important point that contributes to the gravity of CVL is associated with the disorganization of splenic tissue. Dogs that presented positive spleen cultures also showed more disrupted spleen architecture, with a lower concentration of serum albumin and creatinine and higher levels of aspartate aminotransferase. Together, these data suggest that the disorganization of lymphoid tissue in the spleen is linked to more severe clinical presentations of CVL (Lima et al., 2014).

The immunosuppression observed during CVL is also correlated to the occurrence of CD4+ and CD8+ T cell exhaustion and could be responsible for the absence of specific antigen blastogenesis and IFN-γ secretion. Moreover, there was a significant increase in the surface expression of programmed death 1 (PD-1) on T cell populations, mainly in CD8 T cells of symptomatic dogs in comparison to control animals. Using monoclonal antibodies able to inhibit the PD-1 ligand B7.H1 restored CD4^+^ and CD8^+^ T cell function and raised the levels of reactive oxygen species in cocultures of phagocytes and T cells. Consequently, these macrophages showed a reduction in the number of intracellular parasites. The T cell exhaustion resulting from symptomatic CVL may affect the response to vaccination and the efficacy of treatments used to control *L. infantum* (Esch et al., 2013).

Different reports in the literature have associated the parasite burden in different tissues with the host immune response, evaluating pro- and anti-inflammatory cytokines. Generally, there is a consensus that a mixed Th1/ Th2 response is observed at the time of detection of a *Leishmania* infection, despite the prevalence of a Th2 profile. Later in the infection, when the parasite load decreases, e.g., as a result of treatment, the Th1 profile becomes predominant. However, this is not always the case. In a study of 20 infected dogs treated with miltefosine and allopurinol, 80% of animals showed expansion of the parasite load in blood and lymph nodes and low IFN-γ levels at the end of the 9–12 months study period (Manna et al., 2008). This response signals a failure to induce a Th1 response in the majority of treated dogs. Nevertheless, these animals did not show any sign of disease, suggesting they are in an asymptomatic state (Manna et al., 2008). The above highlights the complexity of CVL and the absence of reliable markers of recovery of infected dogs. In another study, quantifying TNF-α, IL-4, and IL-10 in the spleen and liver of dogs naturally infected with *L. infantum*, with or without clinical signs, showed that the animals exhibited higher levels of these cytokines compared to control non-infected dogs (DE F Michelin et al., 2011). Interestingly, the authors found that the liver is the main organ responsible for the production of cytokines during infection. Moreover, TNF-α was positively correlated with parasite burden and could represent a marker for disease infection, with the participation of IL-10 (DE F Michelin et al., 2011).

More recently, the parasite burden and levels of IFN-γ, TNF-α, IL-10, and TGF-β were evaluated in 5 target tissues at 6 and 16 months after infection with *L. infantum* in an experimental canine model (Rodriguez-Cortes et al., 2016). The data showed that the spleen and liver of infected animals exhibited a high parasite density at both time points and produced pro- and anti-inflammatory responses (Rodriguez-Cortes et al., 2016). The popliteal lymph nodes produced IFN-γ both at the beginning of the infection and in the chronic phase. In contrast, an increase in IL-10 and TGF-β expression in these organs was only observed in the chronic phase. Of note, cytokines were absent in the skin, although parasites were detected at 6 months post-infection. Therefore, in the above-mentioned study, the spleen and liver of infected dogs produced diverse cytokines at early times of infection, whereas an anti-inflammatory profile was observed in peripheral tissues at later periods, considered as the chronic phase of infection (Rodriguez-Cortes et al., 2016). Another study of experimentally infected dogs, considered asymptomatic due to the few clinical signs presented, animals were maintained for six years after intradermal infection (Abbehusen et al., 2017). Although few clinical signs were noticed in these animals, most presented parasites in the lymph nodes, spleen and skin and exhibited an increase in IFN-γ, GM-CSF, IL-6, and IL-18 levels and a decrease in TNF, IL-2, and CXCL1 serum concentrations. These results seem to suggest that a persistent activation of the immune system in subclinical infections with *L. infantum* may possibly control parasite growth and limit disease severity (Abbehusen et al., 2017).

Our group studied 70 naturally infected dogs from an endemic area in Bahia, Brazil, grouping the animals according to a clinical score previously described by Manna et al (Manna et al., 2009) with slight modifications. In the group of dogs with severe disease (clinical score >7), we observed a reduction in the levels of serum LTB-4 and PGE-2 and an elevation in chemokine concentrations such as CXCL1 and CCL2 (Solca et al., 2016). Performing ROC curves, we observed that a combination of LTB-4, PGE-2, and CXCL-1 differentiated best among distinct groups of dogs with different clinical scores. Besides that, analysis of the interactome of the different mediators evaluated showed that LTB-4, a lipid mediator, had the highest number of interactions with other cytokines and chemokines in the group of dogs with severe disease. Although IFN-γ and IL-10 were also evaluated, we did not find an important role for these cytokines as markers of disease progression in infected dogs (Solca et al., 2016). Table 2 summarizes the main findings about biomarkers in canine ZVL.

**Table 2:** Biomarkers in canine visceral leishmaniasis.

**Cytokines**	**Chemokines**	**Lipid mediators**	**Mixed molecules**	**Signature**	**References**
IFN- γ, IL-2 and TNF-α				↑ Resistance to infection	Reis et al., 2010; Barbosa et al., 2011; do Nascimento et al., 2013
				↑ Beginning of infection	Rodriguez-Cortes et al., 2016
IL-4, IL-5, IL-13 and IL- 10 TGF-β				↑ Susceptibility to infection ↑ Chronic phase	Reis et al., 2010; Barbosa et al., 2011; DE F Michelin et al., 2011 Rodriguez-Cortes et al., 2016
			IRP2 (iron regulatory protein 2)	↑ Susceptible dogs	do Nascimento et al., 2013
			CD163 (M2 macrophages)	↑ In macrophages from spleen and lymph nodes with ↑ parasite load	Moreira et al., 2016
			PD-1-PDL-1	↑ Symptomatic dog-exhaustion of CD4 AND CD8 T cells	Esch et al., 2013
IFN-γ, GM-CSF, IL-6, and IL-18				↑ Dogs infected by 6 years without clinical signs	Abbehusen et al., 2017
TNF, IL-2	CXCL1			↓ Dogs infected by 6 years without clinical signs	
		LTB-4 and PGE-2		↓ Dogs with severe disease	Solca et al., 2016
	CCL2, CCXL1			↑	

In CVL, the complexity of the immune response and the role played by different mediators during infection by *L. infantum* is clear. Although IFN-γ and IL-10 seem to be important cytokines and are induced during infection, other mediators could be more useful as markers of disease progression or in the assessment of the response to treatment. An important point that deserves consideration is the presence of parasites in different organs of infected dogs, including skin. As such, asymptomatic dogs could represent a good source of parasites to uninfected sand flies contributing to their persistence in endemic areas. These findings indicate that the search for markers of exposure to sand flies is essential for surveillance of endemic areas.

## Biomarkers of vector exposure

In large part, “biomarkers” is a term mostly used to describe characteristics of a disease. However, for vector-borne diseases they can also reference exposure to the arthropod vectors that transmit them. This relatively new discipline of research has gained momentum in recent years and is proving to be of value to various approaches aiming at disease treatment or control (Andrade and Teixeira, 2012; Doucoure and Drame, 2015; Lestinova et al., 2017). In contrast to disease biomarkers, good vector exposure biomarkers may be used as preventative tools that can identify a heightened risk for contracting disease (Carvalho et al., 2015; Ya-Umphan et al., 2017). Further, good biomarkers of exposure may also be used to monitor the expansion or contraction of vector ranges over time, an important tool in light of climate change and its effect on changing the global distribution of disease vectors (Parham et al., 2015; Purse et al., 2015).

The basic requirements that define a good disease biomarker also apply to biomarkers of vector exposure. A reliable biomarker should be specific to a particular vector species (Poinsignon et al., 2008; Teixeira et al., 2010; Ali et al., 2012; Doucoure et al., 2012; Zhao et al., 2015), and recognized by the majority of the exposed or target host population (Souza et al., 2010; Marzouki et al., 2012; Doucoure and Drame, 2015; Mukbel et al., 2016). Additionally, an ideal vector biomarker should be relatively short-lived in the absence of exposure, a desired feature for use in biomonitoring (Clements et al., 2010; Gidwani et al., 2011; Noukpo et al., 2016). For arthropods that transmit pathogens by bite, which represents the majority of disease vectors including phlebotomine sand flies, saliva has been the primary target for biomarkers of vector exposure (Andrade and Teixeira, 2012; Courtin et al., 2015; Doucoure and Drame, 2015).

Most vectors bite mammalian hosts to acquire blood. As such, saliva of vectors has evolved to facilitate blood feeding (Ribeiro and Francischetti, 2003; Lestinova et al., 2017) However, in addition to their physiological effects, several salivary molecules of major disease vectors are immunogenic in humans as well as animal reservoirs and have the potential to become biomarkers of exposure. (Andrade and Teixeira, 2012; Dama et al., 2013a; Abdeladhim et al., 2014; Doucoure and Drame, 2015). Considering that vector control continues to be considered one of the most effective methods to inhibit the transmission of vector-borne diseases, developing immunogenic salivary proteins as biomarkers of vector exposure represent a powerful tool in our arsenal toward their control.

Measuring the antibody response to total saliva, though useful, has serious limitations, mostly due to cross-reactivity of some antigens in various sympatric vectors or host-biting non-vectors (Andrade and Teixeira, 2012; Dama et al., 2013a,b; Doucoure and Drame, 2015; Simo et al., 2017). There are also technical limitations to the use of total saliva preparations as marker of vector exposure including reproducibility and scale-up. This prohibits its consideration for use in large-scale epidemiological surveys. To overcome such obstacles, well-defined salivary antigens or peptides that are specific to a particular vector species, while maintaining a measurable immunogenicity in the target population, are being pursued (Rohousova et al., 2005; Poinsignon et al., 2008; Teixeira et al., 2010; Ali et al., 2012; Zhao et al., 2015; Sima et al., 2016).

### Biomarkers of exposure to vector sand flies

Sand fly saliva consists of a relatively small number of secreted proteins that are injected into the skin in small quantities (Abdeladhim et al., 2014). Nevertheless, sand fly saliva is highly immunogenic and induces a potent cellular and humoral immune response in humans and animals including dogs, known reservoirs of *L. infantum* (Andrade and Teixeira, 2012; Abdeladhim et al., 2014; Lestinova et al., 2017). Though it has been well established that induction of a saliva-specific Th1-biased cellular immunity protects from disease, saliva-specific antibodies have been associated to both protection, for vectors of visceral leishmaniasis (Barral et al., 2000; Gomes et al., 2002; Aquino et al., 2010; Vlkova et al., 2011), as well as an enhanced risk of infection, for vectors of cutaneous leishmaniasis (Mondragon-Shem et al., 2015). To date, we have no clear understanding of the reason behind these contrasting associations.

Whether antibodies to saliva are associated to protection or risk, they remain a good indicator of the rate of exposure to bites of vector sand flies. However, as for other vector-borne diseases, there are more than one man-biting species of sand flies in endemic areas, and sympatric vectors transmitting different types of leishmaniasis are not uncommon (Rohousova et al., 2005; Clements et al., 2010; Teixeira et al., 2010; Marzouki et al., 2012). This increases the risk of cross-reactive antigens between different species of sand flies and decreases from the efficacy of total saliva as reliable biomarkers of vector exposure. For this reason, defined salivary antigens specific to a particular vector species are being developed as markers of exposure in humans and reservoirs (Andrade and Teixeira, 2012; Lestinova et al., 2017). One of the most promising markers of human exposure to vector sand flies is PpSP32, a salivary protein from *Phlebotomus papatasi*. PpSP32 was identified as an immunodominant antigen in a naturally exposed population in Tunisia and was later validated in large-scale studies of endemic populations in Tunisia and Saudi Arabia (Marzouki et al., 2012, 2015; Mondragon-Shem et al., 2015).

### Biomarkers of exposure to bites of *lutzomyia longipalpis*, the principal vector of ZVL in latin america

*Lutzomyia longipalpis*, the main vector of ZVL in Latin America, has a wide distribution range extending from Mexico to Uruguay (Lainson and Rangel, 2005; Brazil, 2013). The domestic dog is considered a main reservoir host and is a major source of sand fly infection and human disease (Lainson and Rangel, 2005; Roque and Jansen, 2014). As such, it is clear that developing reliable biomarkers of exposure to *L. longipalpis* for humans and dogs would provide a useful epidemiological tool for monitoring ZVL in Latin America. Two studies have demonstrated the immunogenicity of *L. longipalpis* saliva in endemic populations, associating a positive saliva-specific antibody response to protection against ZVL (Barral et al., 2000; Aquino et al., 2010). The screening of recombinant proteins representing major secreted salivary molecules in *L. longipalpis* saliva revealed LJM11 and LJM17 as potential markers of vector exposure for both humans and dogs (Teixeira et al., 2010). Both LJM11 and LJM17 belong to the yellow family of salivary proteins that bind biogenic amines blocking their hemostatic-restoring activity during feeding (Abdeladhim et al., 2014). Relevant to their function as biomarkers of exposure, LJM11 and LJM17 are abundant in sand fly saliva and absent from saliva of other common insects such as mosquitoes (Abdeladhim et al., 2014). However, for use as specific markers of exposure to *L. longipalpis* saliva, absence of cross-reactivity with their homologs in saliva of other sympatric sand fly species needs to be demonstrated. Both LJM11 and LJM17 are not recognized by sera of humans bitten by *L. intermedia*, a vector of cutaneous leishmaniasis in Brazil whose distribution commonly overlaps with *L. longipalpis* (Teixeira et al., 2010). Further, only one of two human sera that are strongly reactive to *L. longipalpis* saliva weakly recognized two antigens in *L. intermedia* saliva (Teixeira et al., 2010). A large-scale study further validated the immunogenicity and specificity of LJM11 and LJM17 as markers of exposure to *L. longipalpis* saliva (Souza et al., 2010). Moreover, sensitivity of the assay was enhanced to a level comparable to that against total saliva by using a combination of both LJM11 and LJM17 (Souza et al., 2010).

In contrast to other zoonotic *Leishmania* infections, canids are considered the only reservoir of *L. infantum* infection. As such, developing a marker of vector exposure to *L. longipalpis* for dogs is indicated. LJM11 and LJM17 and two other salivary proteins from *L. longipalpis* saliva, LJL143 and LJL23, were highly immunogenic in dogs (Collin et al., 2009; Teixeira et al., 2010). LJL143, also called Lufaxin, has dual anticoagulant and anti-complement activities (Collin et al., 2012; Mendes-Sousa et al., 2017), while LJL23 is an Apyrase, an inhibitor of platelet aggregation (Teixeira et al., 2010; Abdeladhim et al., 2014). Interestingly, SP01B and SP01, Apyrase homologs from saliva of *P. perniciosus*, one of the primary vectors of *L. infantum* in Europe, were also recognized as markers capable of detecting vector exposure in dogs (Abdeladhim et al., 2014; Lestinova et al., 2017) Moreover, SP03B, a yellow protein from *P. perniciosus* saliva was also identified as a good marker of vector exposure in dogs (Kostalova et al., 2017). However, to our knowledge, the specificity of SP01B, SP01, and SP03B to saliva of *P. perniciosus* have not been tested in areas where other dog-biting sand fly species are prevalent. Though geographically separated, it is interesting to note that molecules belonging to the yellow family of proteins and Apyrases of both *L. longipalpis* and *P. pernicious* were identified as promising candidates for vector exposure to ZVL in both humans and dogs (Teixeira et al., 2010; Lestinova et al., 2017). In addition to their use to assess human and dog exposure to sand fly bites, biomarkers can also be developed for use in sentinel animals that are common around humans, such as chicken or domestic ungulates, as indicators of vector prevalence (Soares et al., 2013; Rohousova et al., 2015).

The above-mentioned defined biomarkers of *L. longipalpis* bites are highly promising, however, considering the richness of the sand fly fauna in Latin America, they require further validation of their specificity against salivary homologs from other dominant man- or dog-biting sand fly species. Having said that, the level of specificity of a biomarker should be primarily indicated by its intended use and by the nature of the endemic area. A biomarker can target a particular vector, several vectors, or simply sand fly bites.

Another significant utility for biomarkers of vector exposure is biomonitoring. For this, knowledge of the kinetics of antibody induction and decay is necessary. Previous studies in humans and dogs have demonstrated that saliva-specific antibodies correlate to biting intensity, fluctuate with the sand fly season and are of relatively short duration, declining significantly after bite cessation caused by sand fly seasonality, use of nets or removal from endemic areas, thus demonstrating their usefulness as tools to measure efficacy of vector control interventions (Hostomska et al., 2008; Clements et al., 2010; Gidwani et al., 2011; Vlkova et al., 2011; Marzouki et al., 2012; Kostalova et al., 2015; Quinnell et al., 2018). Nevertheless, the antibody response to saliva may be species-specific and may change in response to a defined antigen. Therefore, kinetics and duration of antibodies should be investigated for the sand fly vector species/biomarker in question. For *L. longipalpis*, two studies investigated the kinetics of antibody response to total saliva in dogs experimentally (Hostomska et al., 2008) or naturally (Quinnell et al., 2018) exposed to bites. In experimentally-exposed dogs, the level of saliva-specific total IgG antibodies correlated positively to the intensity of bites, increasing rapidly with each weekly exposure and declining significantly within 2 weeks of the last exposure, despite maintaining a low titer up to 6 months post-exposure (Hostomska et al., 2008). In dogs naturally exposed to *L. longipalpis* bites, dogs developed saliva-specific antibodies in 2 months, with antibody levels increasing during high transmission/biting intensity and declining rapidly during low transmission/biting intensity (Quinnell et al., 2018).

To date, we have developed the methodologies that identified several promising makers of exposure to *L. longipalpis* bites. Further studies of the utility of these defined antigens, as biomarkers of exposure to *L. longipalpis* need to be undertaken in natural foci to thoroughly investigate their specificity. Additionally, longitudinal studies to establish the kinetics of antibody development and decline to promising defined biomarkers will establish their value in biomonitoring and may even reveal important associations with risk of, or protection from, ZVL.

## Overall conclusion

The complexity of ZVL and CVL challenges the reliability of a single biomarker to assess disease progression in humans and dogs, respectively. More likely, a combination of distinct inflammatory mediators will be needed to provide a tool that can distinguish relevant states of disease, also defining the role played by these different molecules in the pathogenesis of ZVL.

Despite the considerable progress made in defining important biomarkers for both ZVL and CVL, more studies are indicated, as well as an open dialogue by the scientific community, to reach a consensus for a reliable signature of distinct disease states. Another important point to consider evaluating the different biomarkers in ZVL is the possibility to find new targets to improve the treatment of the disease, increasing the chances of cure and avoiding the fatal outcome of infection. The combination of different drugs directed to several molecules would contribute to obtain an effective treatment for patients. In addition, in the future, such biomarkers may also be of value in assessing the level of protection induced by prophylactic strategies.

Together with well-defined markers of exposure to vector sand flies, such tools could become invaluable to evaluate response to treatment, and success of interventions among others.

## Author contributions

CB and SK have equal participation to write this review.

### Conflict of interest statement

The authors declare that the research was conducted in the absence of any commercial or financial relationships that could be construed as a potential conflict of interest.
